# A hybrid implant combining a macroporous device with immunoprotective microcapsules for cell therapy applications: A conceptual in vitro study

**DOI:** 10.1016/j.mtbio.2025.101574

**Published:** 2025-02-27

**Authors:** Adam Stell, Vijayaganapathy Vaithilingam, Sami G. Mohammed, Rick H.W. de Vries, Denise F.A. de Bont, Eelco J.P. de Koning, Aart A. van Apeldoorn

**Affiliations:** a Department of Cell Biology–Inspired Tissue Engineering (cBITE), MERLN Institute for Technology Inspired Regenerative Medicine, Maastricht, the Netherlands; b Department of Internal Medicine, Leiden University Medical Center (LUMC), P.O. Box 9600, 2300 RC, Leiden, the Netherlands; c LUMC Transplantation Center, Leiden University Medical Center, Leiden, the Netherlands

**Keywords:** PVDF, Macrodevice, Microencapsulation, Alginate, Hybrid implant, Cell therapy

## Abstract

Cell encapsulation strategies including macro- and micro-delivery devices have been widely used in cell therapy due to their ability to provide immunoprotection to the transplanted cells. While both strategies can provide mechanical and physiochemical support for maintaining cell survival and function, they each have their limitations. In this study, we report the design and fabrication of a hybrid implant combining the advantages of both macro- and micro-cell delivery devices. The hybrid implant comprises a microwell-array macroporous device fabricated from non-degradable clinically approved polyvinylidene fluoride (PVDF) combined with immunoprotective alginate microcapsules. The microwell design provides a vessel to retain individual microcapsules, while the pores enable unhindered mass transport of nutrients and oxygen to the encapsulated cells and support vascular ingrowth. We show that both rodent pseudoislets and primary human islets maintain their viability and function inside the hybrid implant in a proof-of-concept study. Mechanically, it is strong and flexible suitable for surgical handling and for eventual retrieval for replacement. The hybrid implant also supports the growth of human umbilical vein endothelial cells (HUVEC) across its surface allowing *in vitro* “prevascularization”, which can potentially accelerate blood vessel formation in poorly vascularized transplantation sites such as the subcutaneous space. In conclusion, the hybrid cell delivery device, which confers immunoprotection and allows prevascularization, can act as a protective container during surgical handling and retrieval of microencapsulated cells opening a wide range of cell therapy applications including stem cells.

## Graphical abstract

**Figure:**
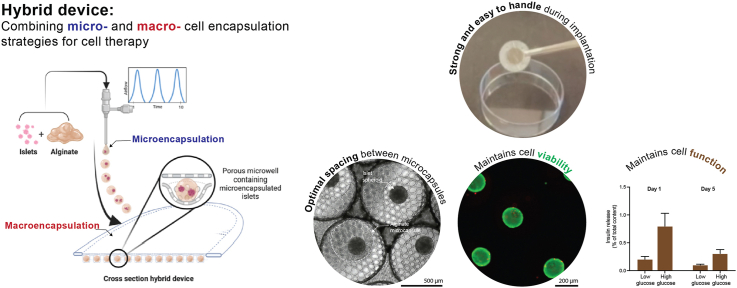

## 1 Introduction

Cellular therapies employing both primary and stem cells are being studied in clinical trials and fundamental research to treat a wide variety of disorders including cancer, neurological, endocrine, cardiovascular, and metabolic disorders [[1], [2], [3], [4], [5], [6]]. Cell therapy treatment involves either direct infusion of primary autologous cells or the use of engineered cells to repair damaged tissue or secrete bioactive factors to achieve a desired therapeutic effect [[7], [8], [9]]. Direct injection of cells either systemically or locally can lead to poor engraftment resulting in undesired therapeutic outcomes or may raise safety concerns if, for example, stem cell-derived immature differentiated cells end up in off-target tissues [10,11]. These issues can be resolved using so-called encapsulation strategies to deliver and constrain cells to a desired transplantation site by means of a biomaterial-based cell delivery device, thereby enhancing cell retention, engraftment, and function [12,13]. Typically, encapsulation involves the use of a dedicated tailor-made delivery device to retain the cells within a closed carrier that protects them from the host immune system while allowing for optimal diffusion of nutrients and oxygen. Ideally, such cell delivery devices should provide sufficient mechanical support for easy handling during implantation, and subsequent retrieval when replacement is needed over time, without evoking a strong foreign body response at the implantation site of interest [14]. Moreover, immunoprotective delivery devices allow for the transplantation of allogeneic, stem cell-derived or even xenogeneic cells without the need for immunosuppression since the recipient's immune cells are blocked outside avoiding direct interaction with the delivered cells inside the device.

Generally, cell delivery devices are of two types a) macrodevices, which contain a large number of cells in one single device, and b) microcapsules, which consist of small hydrogel droplets in which single cells, or cell aggregates are captured. Both cell delivery strategies have their own merits and have been shown to perform well in preclinical models, but also have some limitations for use in an upscaled clinical application [[15], [16], [17]]. Small hydrogel microcapsules for cell delivery have the advantage that mass transport of essential nutrients, oxygen, and amino acids can be easily controlled by tuning the hydrogel properties, while their size allows for a short diffusion pathway to and from the cells. A large number of microcapsules is needed to reach the desired clinical effect; however, handling and retrieval of the microcapsules and control over their precise location during long-term placement in the body remain challenging due to their small size [18].

Macrodevices can be made from clinically used biomaterials and are able to contain a large number of cells in one location, thus the confinement and retrieval of cells is relatively easy [19]. However, macrodevices suffer from long diffusion pathways and bulky cell aggregation on the inside, leading to suboptimal mass transport of nutrients and oxygen to and from the encapsulated cells leading to less favorable transplant outcomes [20]. In essence, where large-scale macrodevices suffer from a relatively small surface area for mass transfer, cumbersome retrievability after implantation remains an issue with microcapsules in the event of complications, loss of function, and replacement. Ideally, a cell delivery device should encompass the best of both macro and micro devices, a design that can harness the positive aspects of both delivery strategies in one hybrid implant excluding the negative aspects.

In this study, we report the design and fabrication of a hybrid implant based on a microwell array thin polymer film macroporous design containing individual immunoprotective cells-containing hydrogel microcapsules. This hybrid design consists of a thin film open macroporous structure to promote mass transfer, and vascular ingrowth on the outside, while physically separating a multitude of individual microcapsules on the inside due to its microwell array design. The porous macrodevice envelope is fabricated from an ultra-thin sheet of laser-drilled porous non-degradable clinically approved biomaterial, polyvinylidene difluoride (PVDF), which is subsequently micro thermoformed into arrays of well-defined microwells which are homogeneously distributed across its surface. In parallel, cells-containing ultrapure alginate microcapsules are produced by an air-driven droplet generator and subsequently seeded into the macrodevice capturing individual microcapsules in separate microwells allowing for a uniform distribution throughout the hybrid implant. As a proof of concept, both microencapsulated primary human islets, and pseudoislets produced from an insuloma cell line as a model for beta cells, seeded into the hybrid device maintained their respective viability and function *in vitro* over time. In addition, we demonstrated the prevascularisation potential of the hybrid implant by seeding human umbilical vein endothelial cells on the surface of the hybrid device, which adhere and proliferate to form an endothelial cell layer within the microwells.

## 2 Materials and methods

**Fig. 1:**
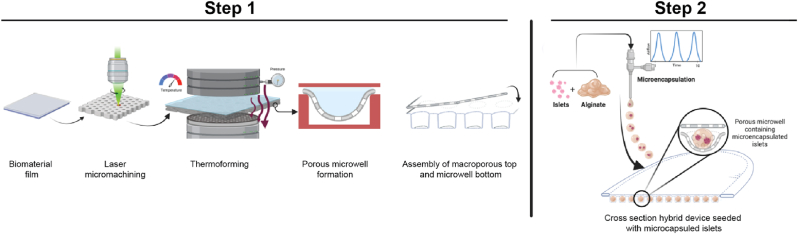
**Schematic overview of the hybrid device fabrication.** Step 1 includes the fabrication and assembly process of the thin film microwell device (macrodevice). Step 2 includes the microencapsulation of cells and seeding into the macrodevice.

For clarity, the manufacturing process of the hybrid implant is schematically depicted in Fig. 1. Details regarding design and fabrication of the open microwell-array macrodevice and the generation of microcapsules for encapsulation of islets or pseudoislets are described below.

### 2.1 Microwell-array macroporous device fabrication and characterization

#### 2.1.1 Polymer thin film fabrication and laser micromachining

A 15 % polyvinylidene difluoride (PVDF) (medical grade Kynar 720, Solvay) solution was solvent casted, as previously described to create solid thin films of PVDF [21]. Briefly, a 15 % (w/w) polymer solution was prepared in dimethyl formamide (DMF, Sigma-Aldrich). Subsequently, an automatic film caster (Elcometer K4340M12) located within a humidity-controlled box was equipped with a dedicated glass plate and preconditioned at a temperature of 100 °C at 10 % humidity. The 15 % PVDF-DMF solution was then casted onto the glass plate using an applicator (Elcometer K0003530M005) set at a gap distance of 250 μm running over the polymer solution at a constant speed of 5 mm/s to homogeneously spread the dissolved polymer over the surface of the glass plate. After solvent evaporation the polymer film was dried overnight under continuous nitrogen gas flow, resulting in a 15 μm thick solid PVDF film. Polymer films were washed in de-mineralized water overnight to remove any remaining solvent residue and air-dried afterwards. To create a porous film, a preprogrammed pattern of pores was made by femtosecond laser micromachining using an UV pulsed laser at a frequency of 25 kHz (in collaboration with Veldlaser Innovations B.V., ‘S-Heerenberg, NL). PVDF films used for the microwell array bottom of the device were patterned with pores having a pore size of 25 μm and 50 μm pitch to compensate for the stretching during microthermoforming, while polymer films used as lids were patterned with a pore size of 40 μm and 100 μm pitch.

#### 2.1.2 Microthermoforming of porous thin films

After laser micromachining PVDF thin films, microwell-arrays were fabricated by micro-thermoforming in a dedicated stainless-steel mold as described previously [22]. A 50 mm by 50 mm metal mold was designed to contain ∼90 through-holes per device in a circular pattern. Each hole was 800 μm in diameter, while the diameter of the resulting device was around 0.9 cm. During thermoforming, the porous laser micromachined PVDF films are placed onto the mold while a 560 μm thick polyethylene film is placed on top of the PVDF film in a hydraulic hot press (Atlas manual hydraulic press, Specac) and left to heat up for 2 min at 85 °C. The pressure is then subsequently increased to 50 kN to form the microwell array. After 10 min of compression and cooling down, the microwell array is removed from the hydraulic press and submerged in ice cold ethanol for 5 min to enable removal of the microthermoformed film from the mold.

#### 2.1.3 Fabrication of support rings

2 g of PVDF pellets were loaded onto a stainless-steel mold made from a 10 by 10 cm plate engraved with a 9 cm diameter disc, with a depth of 200 μm, and placed in an Atlas manual hydraulic hot press (Specac). The hot press and PVDF containing mold are preheated for 1 min at 180 °C prior to compression. After preheating the pressure is increased and maintained at 20 kN for 1 min. After cooling down, the resulting 9 cm diameter 200 μm thick PVDF disc is removed from the hydraulic press and mold. Subsequently, support rings were cut from the PVDF disc with a knife based cutting plotter (Silhouette Cameo 4) to create a PVDF ring matching the device dimensions which can be fit to the rim to act as mechanical support structure.

#### 2.1.4 Device assembly and ultrasonic welding

A stainless-steel custom-made ultrasonic welding guide was used in which all device components can be placed to exactly align all components prior to welding to ensure reproducible and accurate assembly of the device. The thermoformed bottom half is first placed in the stainless-steel holder followed by a support ring and then the porous lid. Finally, a Teflon cover plate for guiding the ultrasonic welding process is placed on top. All device components are welded at the rim of each device using a manual Branson LPX US welding station at 75 % amplitude, leaving only a small non-welded entry port for future seeding of microcapsules.

#### 2.1.5 Mechanical tensile testing

Mechanical properties of PVDF films were determined by tensile testing using a 45 kN load cell on a Electroforce 3230-ES Series III test instrument (UK). Samples were cut using a standardized dog-bone mold and had an effective area of 15 × 2 mm between clamps. The ramp speed was set at 0.015 mm/s for solid PVDF films, and 0.15 mm/s for laser drilled and thermoformed sheets. Each condition was tested four times. From the stress-strain curves, peak strain, failure stress, failure strain and Young's modulus were calculated using MATLAB (software version R2020b).

#### 2.1.6 Pore size determination and microwell dimension

As part of quality control, the dimensions and shape of the pores and microwells, in random test samples, were examined by scanning electron microscopy (SEM) to check for any defects or malformations which might have occurred during microthermoforming. Device samples are attached to metal pin stub mounts (Electron Microscopy Sciences, Hatfield USA), gold coated using a Quorum SC7620 sputter coater (East Sussex, UK) and observed by SEM. Electron micrographs were made using a JSM-IT200 SEM (JEOL, Tokyo, Japan). Microwell and pore shape and dimensions (depth, width, diameter) were measured with JSM-IT200 image analysis software, measurements were collected and statistical analysis was done using Prism/GraphPad software.

### 2.2 Cell culture

#### 2.2.1 Rat INS1E cells

Rat INS1E insulinoma cells (AddexBio, USA) were cultured in RPMI 1640 (Gibco) supplemented with 10 % (v/v) fetal bovine serum, 100 U/mL penicillin and 100 mg/mL streptomycin, 1 mM sodium pyruvate, 10 mM HEPES and 5 mM glucose. For cell expansion, 2-mercaptoethanol was added fresh to the culture to a final concentration of 1 μl/mL medium and medium was refreshed every 2–3 days.

#### 2.2.2 Mouse MIN6 cells

Mouse MIN6 insulinoma cells were cultured in DMEM (Gibco) supplemented with 10 % (v/v) fetal bovine serum, 100 U/mL penicillin and 100 mg/mL streptomycin, and 5 mM glucose. For cell expansion, 2-mercaptoethanol was added fresh to the culture to a final concentration of 1.2 μl/mL medium and medium was refreshed every 2–3 days. MIN6 cells were kindly provided by Dr. Philippe Halban (University of Geneva, Switzerland) with permission from Dr. Jun-ichi Miyazaki (University of Osaka, Japan) who produced the maternal cell line.

#### 2.2.3 Rodent pseudoislets

Pseudoislets were produced using non-adherent agarose microwell chips as described before (24). Briefly, microwell chips containing 800 microwells with a diameter of 400 μm were fabricated in a sterile manner by pouring a 3 % (w/v) UltrapureTM agarose (Invitrogen, Bleiswijk, the Netherlands) solution onto molds of polydimethylsiloxane (PDMS) placed in 6 well plates. After the agarose solidified, the molds were removed and the resulting agarose microwell containing discs transferred to 12 well tissue culture plates an immersed in sterile phosphate buffered saline and stored at 4 °C until use. Before cell seeding, the agarose microwells were pre-incubated with INS1E (or) MIN6 culture media overnight at 37 °C. For aggregate formation, single INS1E (or) MIN6 cells are resuspended in fresh medium, and then seeded onto agarose chips at a concentration of 400,000 cells per agarose microwell chip which is equal to ∼500 cells per microwell. Immediately after seeding, the agarose microwells disc are centrifuged for 1 min at 300 G to concentrate the cells in the bottom of microwells. Subsequently respective cell culture medium was added to the well plate and refreshed every 1–2 days. The INS1E (or) MIN6 pseudoislets were cultured up to 3 days after seeding before removal from the microwells by upside down centrifugation (1 min, @ 200 G) to stabilize them for use in subsequent microencapsulation studies.

#### 2.2.4 Primary human islets

Human islets were kindly provided by the human islet isolation and transplantation center at the Leiden University Medical Center (LUMC, Leiden, the Netherlands). Human islets not deemed suitable for clinical were used in these experiments in accordance with Dutch law. Briefly, human islets were isolated from cadaveric pancreas and cultured in CMRL-1066 media as described previously [23]. A dithizone staining was performed before shipment to determine islet purity and was found to ∼80 %. Upon receiving the islets, the media was removed after centrifugation (180×*g* for 2 min), followed by resuspension in fresh CMRL-1066 medium (Pan Biotech, Aidenbach, Germany) supplemented with 10 % (v/v) FBS, 10 mM HEPES, 1 % Penicillin-Streptomycin and 10 μg/mL Ciprofloxacin. Islets were then allowed to recover for 24 h in free floating conditions before use in subsequent microencapsulation studies. Informed consent was obtained from all subjects and/or their legal guardian(s) prior to organ retrieval. Experiments performed with human islets in this paper were done in accordance with the relevant guidelines and regulations for research purposes.

#### 2.2.5 Human umbilical vein endothelial cells

Human umbilical vein endothelial cells (HUVEC) (Lonza) were cultured in endothelial cell medium, (EGM-2) (BulletKit, Lonza, Cat. No. CC-3162) supplemented with 2 % fetal bovine serum (FBS) as described previously [24,25]. HUVECs were harvested for seeding onto hybrid devices at passages between 3 and 10.

### 2.3 Microencapsulation and macroporous device seeding

#### 2.3.1 Alginate microcapsules

The formation of alginate microcapsules was carried out using an air-driven droplet generator (Nisco VarJ1, Zurich, Switzerland) as described previously [26]. Briefly, before the encapsulation procedure, aliquots of MIN6 (or) INS1E pseudoislets and human islets were collected and mixed thoroughly with 2.2 % w/v ultrapure sodium alginate (>60 % G sodium alginate Pronova, UPMVG, Novamatrix, FMC Biopolymer, Norway) in a 1:8 ratio (cells: alginate) in a 50 ml falcon tube. Subsequently, the alginate cell mixture was then extruded through the nozzle of the droplet generator with an airflow of 5 L min-1 leading to the formation of uniform droplets. The droplets were collected in a sterile 145 mm petri dish containing 50 ml barium chloride (BaCl_2_) (20 mM) gelling solution to crosslink the alginate molecules after which cell containing microcapsules were formed. The microcapsules were left to incubate in the BaCl_2_ solution for 2 min after which they were collected and washed five times with Dulbeco's phosphate buffered saline (DPBS) to remove any excess barium. After washing, the microcapsules were allowed to settle down, and the remaining supernatant was discarded. The microencapsulated cells were then cultured for a 24 h in appropriate media before seeding into the macroporous device.

#### 2.3.2 Seeding into macrodevice

For macroporous device seeding, a 200 μl modified pipette tip (tip removed to create a bigger opening) was used to aspirate microcapsules and seed them into the device via the inlet port in between the top and microwell array containing the bottom film. Briefly, ∼50 microcapsules were resuspended in 200 μl of culture medium, and slowly dispensed into the device. While seeding, the pipette tip is slowly moved from side to side to distribute the microcapsules evenly throughout the device. After seeding, a hand-held mini heat sealer (Ortable) was used to seal the inlet as the final assembly step of the hybrid implant.

### 2.4 Encapsulated cell characterization and function

#### 2.4.1 Cell viability

The viability of microencapsulated cells within the hybrid implant was assessed using the fluorescent dyes Calcein AM (for live cells) and Ethidium homodimer-1 (for dead cells) (Thermo Fisher Scientific, The Netherlands). Briefly, each hybrid implant was carefully opened and flushed with DPBS to retrieve the microcapsules into a sterile tissue culture dish. Aliquots of encapsulated cells were randomly collected from the tissue culture dishes with a Gilson P 1000 μl pipette under sterile conditions. They were washed twice by suspending them in 0.5 ml PBS followed by careful removal of the supernatant and discarded. Subsequently, a solution of 4 ml of PBS and 2.5 μl of calcein AM and 5 μl of ethidium homodimer-1 were prepared and 1 ml of this solution added to each sample and incubated for 30 min in darkness. The microencapsulated cells were then visualized under a fluorescence microscope (Nikon Eclipse E600) and analyzed using FIJI software (https://fiji.sc/). Live/dead images were quantified as described previously [27], and the percentage of green (live cells) to red (dead cells) was estimated in 25 microcapsules. Microencapsulated free floating MIN6 (or) INS1E pseudoislets and human islets served as controls.

#### 2.4.2 ATP measurement

For ATP measurements, aliquots of microencapsulated cells retrieved from the hybrid implant were washed twice by resuspending them in 0.5 ml PBS, discarding the supernatant and resuspending again in 100 μl of PBS followed by addition of 100 μl of CellTiter-Glo® 3D Reagent (Promega, Madison, United States). The contents were mixed vigorously for 5 min to induce cell lysis and the plates allowed to incubate at room temperature for a further 25 min to stabilize the luminescent signal. After 30 min of incubation, luminescence was measured on a microplate reader (CLARIOstar) and the amount of ATP measured which signals the presence of metabolically active cells.

#### 2.4.3 Glucose-stimulated insulin secretion (GSIS) functional assay

GSIS assay is used to determine the insulin secretory ability of microencapsulated MIN6 (or) INS1E pseudoislet and human primary islet and hence assess the functional capacity of the hybrid implant. For the assay, Krebs's buffer stock solution (25 mM HEPES, 115 mM NaCl, 24 mM NaHCO_3_, 5 mM KCl, 1 mM MgCl_2_· 6H_2_O, 2.5 mM CaCl_2_ · 2H_2_O, 0.2 % bovine serum albumin in sterile water) was supplemented with glucose, forming either a high (16.7 mM) or low (1.67 mM) glucose solution. Hybrid implants containing microencapsulated pseudoislets (or) human islets (∼50/device) were removed from media and placed in 12-well plates. Free floating microencapsulated pseudoislets (or) human islets served as controls and were handpicked and moved to cell culture inserts (∼50/insert), which were then placed in 24-well plates. All samples were washed three time and incubated for 1 h in a low glucose solution at 37 °C to wash out all remaining insulin. Afterwards, all samples were incubated for another 1 h in a low glucose solution (1.67 mM) followed by 1 h of incubation in a high glucose solution (16.7 mM). After each incubation step, an aliquot of glucose solution was transferred to a 96-well plate and stored at −30 °C until an insulin ELISA assay was performed. For total insulin content, the samples were placed in an acid ethanol solution (1.5 % HCl in 70 % ethanol) and incubated overnight at 4 °C. Acid ethanol supernatants were collected the following day and stored at −30 °C until the insulin ELISA assay. ELISA kits for human and rat insulin (Mercodia, Uppsala, Sweden) were used to determine the insulin concentration after GSIS according to the manufacturer's instruction. The optical density of the samples was read at 450 nm with a spectrophotometric plate reader (CLARIOstar Plus, BMG Labtech). Samples were diluted with Krebs buffer when needed. Finally, the stimulation index (SI), a measure of islet functionality, was calculated by dividing the insulin secretion in high glucose solution by corresponding insulin secretion in the low glucose incubation step.

### 2.5 Prevascularization of the macroporous device

To assess if the macroporous device films support prevascularization, human umbilical vein endothelial cells (HUVEC) were cultured on device films and assessed for attachment and viability with scanning electron microscopy and live/dead staining, respectively. Varied macroporous device components were immersed in a suspension of HUVECs (1 × 10^4^ cells) in a 12-well plate and then placed in the incubator at 37 °C and 5 % CO_2_ for 14 days with intermittent media changes performed every 2–3 days. After 14 days, the morphology of HUVEC cultured on macrodevices was examined by scanning electron microscopy (SEM). Briefly, the devices were washed twice with PBS (pH = 7.4) and fixed in 2.5 % glutaraldehyde for 12 h at 4 °C, then washed again with PBS (pH = 7.4). The samples were dehydrated by exposing to an increasing ethanol series (30, 50, 70, 90 and 100 %) for 10 min each. Critical point drying was done using a EM CPD300 critical point dryer (Leica). All samples were gold coated using a Quorum SC7620 Sputter Coater (East Sussex, UK) and observed by SEM. Electron micrographs were made on a JSM-IT200 SEM (JEOL, Tokyo, Japan). Briefly, microporous lid and microwell bottom films of the hybrid device were fixed onto the bottom of two wells of a 12-well plate by placing a metal ring on top. To further promote the attachment of the HUVECs onto the surface of the films, the films were coated with 0.2 % (v/v) gelatin for 15 min at room temperature and allowed to dry. Next, 2.5 × 10^4^ HUVECs were seeded on top of the films and cultured at 37 °C and 5 % CO_2_ for 14 days while refreshing media every 2–3 days. The films were, then, washed with DPBS once and stained with live/dead staining as mentioned in section 2.4.1.

### 2.6 Statistical analysis

All results were presented as mean ± Standard Error of the Mean (SEM). Statistical analysis was performed using GraphPad PRISM 8 software. Group comparisons were performed using one-way analysis of variance (ANOVA) with a Tukey's post hoc test after assessing the assumptions of equality of variance (Brown-Forsythe test) and normality (Shapiro-Wilk test). If the assumption of normality was not validated, a Kruskal-Wallis test in combination with Dunn's test were used. Direct comparison between two groups was done by an unpaired *t*-test after assessing the assumptions of equality of variance and normality. Welch's correction was used for t-tests if the assumption of equality in variance was violated. A Mann-Whitney test was performed if both assumptions of equality of variance and normality were violated a p-value <0.05 was considered statistically significant.

## 3 Results

### 3.1 Fabrication and characterization of microwell array thin-films

**Fig. 2:**
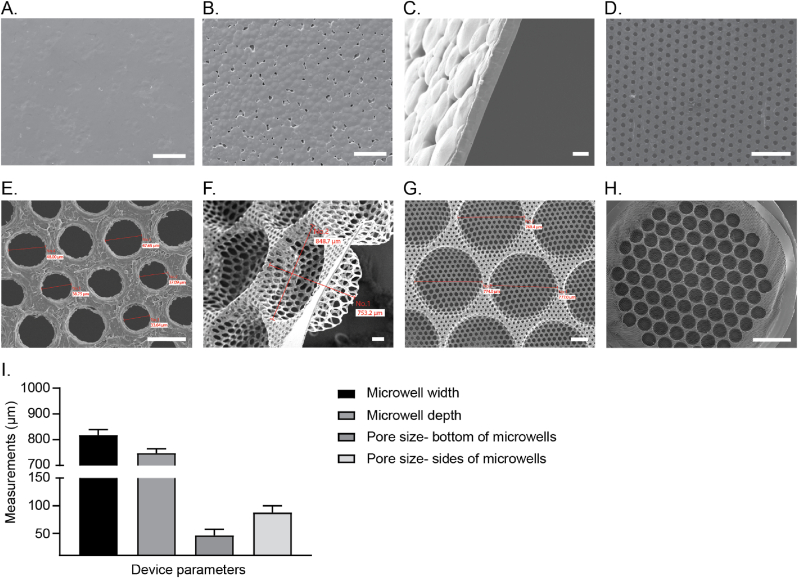
**Scanning electron microscopy images of the polymer films and various stages of microfabrication to create porous thermoformed microwell-arrays macrodevice.** Images of solvent-casted PVDF (A–C) followed by laser-drilled PVDF films (D) and thermoformed to generate microwells (E–H). Views are glass side (A); air side (B) (scale bar = 100 μm); cross-section (C) (scale bar = 10 μm); and laser-drilled pores bottom film (D) (scale bar = 200 μm). Overview of a microwell array showing multiple equally sized and shaped microwells (E) (scale bar = 50 μm); thermoformed microwells (F) (scale bar = 200 μm); cross section of thermoformed microwells (G) (scale bar = 100 μm); and pores at the bottom of microwells (H) (scale bar = 2 mm). Quantification of average well size, width, depth, pore size in the bottom of microwells, and pore size at the sidewalls of microwells in PVDF films after laser micromachining and micro-thermoforming (I). Data are represented as mean ± SD (n = 5–9 microwells).

Casting a 15 % (w/v) PVDF-DMF solution using an automatic film caster equipped with a 250 μm casting knife gap resulted in 15 μm thin dense PVDF films following evaporation of the solvent (Fig. 2A–C). Subsequently, laser micromachining the PVDF films resulted in a regular pattern of equally sized and spaced pores across the film surface (Fig. 2D). The bottom film of the macrodevice was patterned with pores of 25 μm diameter and a pitch of 50 μm prior to thermoforming, while the top film with pore size of 40 μm and pitch of 100 μm. After microthermoforming of the bottom film to create a microwell array, the pores were stretched anisotropically; pores situated at the bottom of the well displayed a round shape with a pore size of 46.1 ± 11.2 μm (Fig. 2E and I) and pores located at the sides of microwells were elliptical shaped along the sidewalls and displayed an average diameter of 87.6 ± 14.2 μm (Fig. 2F and I). The resulting microwells had an average well diameter of 818.2 ± 21 μm and depth of 747.8 ± 16.7 μm, and an average pore density of 205 ± 3 per well (Fig. 2G–I).

**Fig. 3:**
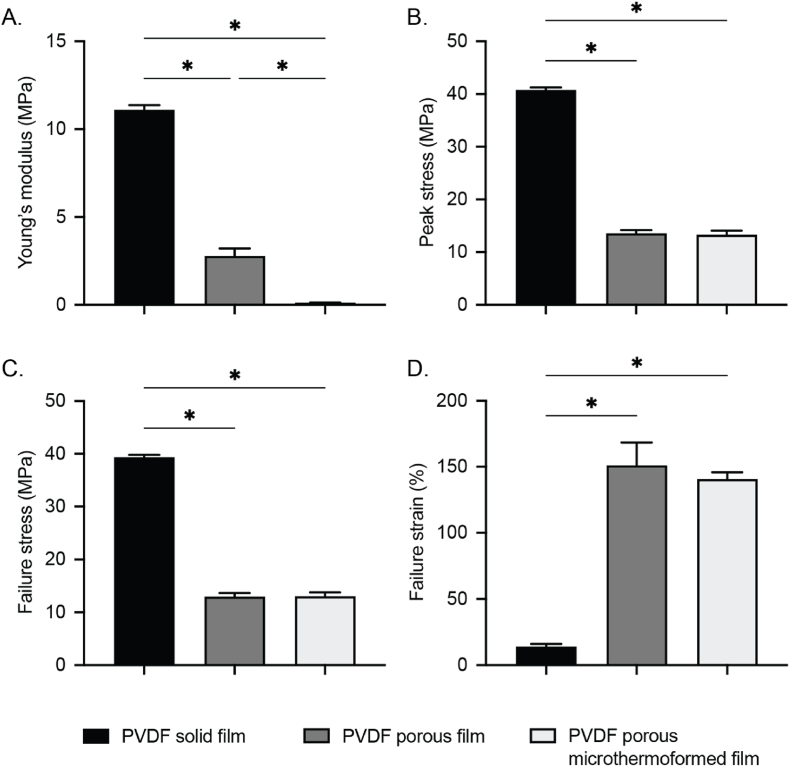
**Mechanical properties of PVDF films at various stages of macrodevice fabrication.** Young's modulus (a measure of material stiffness) (A), Peak stress: ultimate strength (B), Failure stress: force used to break the material (C), and Failure strain: percentage that the material can stretch under load (D). Data are presented as mean ± SD. Significant differences are depicted with asterisk, p < 0.05 using a one-way ANOVA (n = 4).

Tensile tests were done to observe any changes in the mechanical properties of PVDF films at various stages of device fabrication (laser micromachining and microthermoforming). Dog bone-shaped PVDF specimens were prepared according to the international standard (i.e., ASTM: D638) for tensile testing, after which they were deformed with increasing tensile load applied along the longitudinal axis of the material at a constant rate. As shown in Fig. 3A–C, Young's modulus, peak stress, and failure stress values of laser-drilled porous PVDF films dropped significantly compared to untreated solid films (11.10 ± 0.27 vs 2.77 ± 0.43; 40.78 ± 0.45 vs 13.57 ± 0.60; and 39.36 ± 0.44 vs 12.98 ± 0.68, respectively) (Supplementary Fig. 1). However, the failure strain of these porous films significantly increased compared to the untreated solid counterparts (151.05 ± 17.30 vs 14.09 ± 2.02, respectively) (Fig. 3D). Furthermore, microthermoforming porous PVDF films led to a further drop in Young's modulus (Fig. 3A) while the other mechanical test parameters remained similar to the porous films (Fig. 3B–D).

### 3.2 Macroporous device assembly and loading with microencapsulated cells

**Fig. 4:**
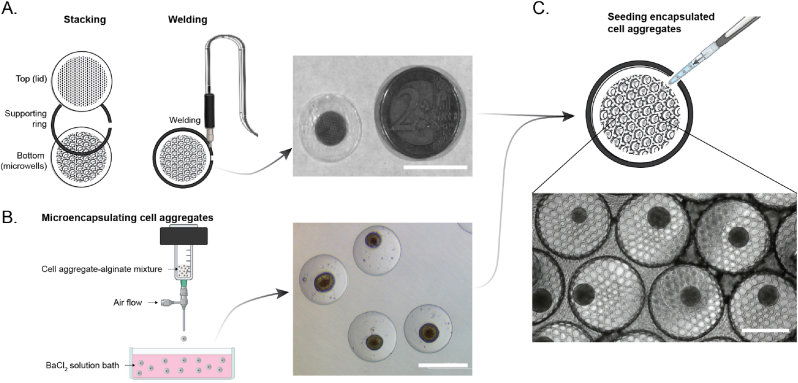
**Device assembly and seeding with microencapsulated islets.** A) Assembly and welding of the microporous top film, the support ring, and bottom film (including microwells) by ultrasonic welding to produce a hybrid implant (∼240 μm thickness; 19 mm diameter; and ∼85 microwells) (scale bar = 20 mm). B) Cell aggregates (pseudoislets or human islets) were mixed with ultrapure sodium alginate and extruded through the nozzle of the droplet generator with airflow to produce microencapsulated cells (∼500 μm) (scale bar = 500 μm). C) Microencapsulated cells seeded into the hybrid device using a wide bore pipette tips, where each microcapsule confined into the microwells of the hybrid implant (scale bar = 500 μm).

The top and bottom films of the macrodevice and a support ring in between were welded together with high-frequency ultrasonic welding at the rim as illustrated in Fig. 4A. A small part was left unwelded to serve as an inlet for seeding microcapsules into the assembled device. The support ring provided strength and structural support to the device to prevent folding and for easy handling during cell seeding, implantation and retrieval (Supplementary movie 1 and 2). Overall, the macrodevice had an outer diameter of 19 mm with the seal at 17 mm diameter on a 2 mm wide flat support ring; and the microwell-imprinted area of 12 mm diameter containing ∼85 microwells. An air-driven droplet generator was used to encapsulate both rodent pseudoislets and human islets alike to generate barium alginate microcapsules. The microcapsules produced were uniform in size with a diameter of ∼500 μm (Fig. 4B). After washing with PBS, ∼50 microcapsules were seeded into a macroporous device through the inlet using a wide-bore pipette tip. After seeding, the inlet is closed using a hand-held heat sealer. The resulting hybrid implant contained approximately one microcapsule per microwell (Fig. 4C and Supplementary movie 2).

The following are the Supplementary data related to this article:

Video 1 Video 1

Video 2 Video 2

### 3.3 Viability and function of pseudoislets within the hybrid implant

**Fig. 5:**
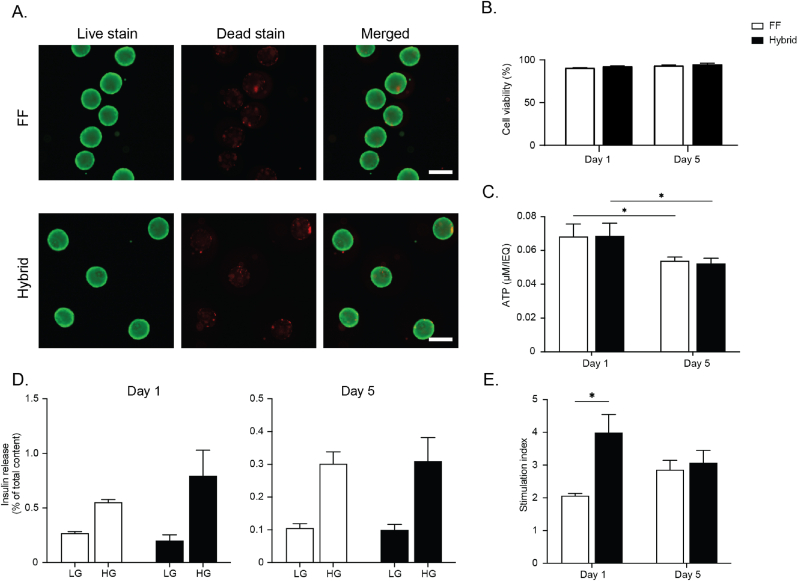
**Viability and function of INS1E pseudoislets in the hybrid implant.** A) Representative live/dead staining images of microencapsulated pseudoislets, free floating (FF) vs hybrid at day 1. (scale bar = 200 μm). B) Percentage of cell viability from the live/dead staining images (mean ± SEM; n = 25 islets). C) The relative ATP amount in both conditions are depicted as per islet equivalent to compensate for variability in islet size indicative of the average number of metabolically active cells in both FF and hybrid implant (mean ± SEM, n = 5, student-t-test ∗p < 0.05). D) Glucose stimulated insulin secretion (GSIS) of microencapsulated pseudoislets FF vs hybrid at days 1 and 5. (LG = low glucose, 1.7; HG = high glucose, 16.7 mmol/l) E) Stimulation indices derived from the glucose stimulated insulin secretion test (mean ± SEM, n = 5, student-t-test ∗p < 0.05).

After assembly of the outer envelope of the hybrid implant, we next determined whether encapsulated rodent pseudoislets cultured inside the hybrid implant remained viable and functional *in vitro*. Several hybrid implants were seeded with ∼50 microcapsules containing rat INS1E pseudoislets, cultured, and evaluated at different time points for viability, ATP, and beta cell functionality, that is insulin secretion, in response to changing glucose concentrations. Microcapsules seeded within the hybrid implant were evenly distributed with each microcapsule residing in one microwell. Live/dead staining revealed microencapsulated rat pseudoislets within the hybrid implant maintained their viability for at least 5 days similar to free-floating controls. There was no significant difference in the viability of encapsulated pseudoislets in the hybrid implant on days 1 and 5 of culture compared to free-floating controls (hybrid vs free-floating: 92.5 ± 0.64 % vs 90.6 ± 0.38 % at day 1; 94.73 ± 1.40 % vs 93.5 ± 0.57 % at day 5) (Fig. 5A and B). ATP measurements support these results, where the ATP levels of free-floating encapsulated pseudoislets were similar to those seeded within the hybrid implant both on days 1 (0.068 ± 0.003 vs 0.069 ± 0.003μM/IEQ) and 5 (0.053 ± 0.001 vs 0.052 ± 0.001μM/IEQ) (Fig. 5C) respectively. Next, encapsulated rat INS1E pseudoislets were subjected to a glucose-stimulated insulin secretion (GSIS) assay to evaluate the functionality of encapsulated beta cells within the hybrid implant. Free-floating microencapsulated pseudoislets served as controls. Both hybrid implant and free-floating controls showed a characteristic low-high insulin release pattern in response to glucose stimuli, indicative of normal beta cell function (Fig. 5D). On day 1, encapsulated rat pseudoislets within the hybrid implant exhibited a better insulin secretory behavior with a significantly higher stimulation index (3.99 ± 0.55) compared to the free-floating controls (2.07 ± 0.07) (Fig. 5E). The effect was maintained until day 5 with pseudoislets in the hybrid device exhibiting a higher stimulation index (3.07 ± 0.38) compared to the free-floating counterparts (2.86 ± 0.28) albeit not statistically significant (Fig. 5E). Similar results were obtained using another beta cell model derived from mouse, namely MIN6 pseudoislets, following microencapsulation and seeding into the hybrid implant. Microencapsulated MIN6 pseudoislets in the hybrid implant maintained high cell viability both on days 1 (91.43 ± 0.93 %) and 5 (95.13 ± 1.45 %) of culture (Supplementary Figs. 2A and B). As expected, there was no significant difference in the ATP levels of microencapsulated MIN6 pseudoislets seeded within the hybrid implant on days 1 and 5 (0.067 ± 0.002 and 0.063 ± 0.002 μM/IEQ, respectively) (Supplementary Fig. 2C). GSIS assay further showed that microencapsulated MIN6 pseudoislets within the hybrid implant maintained their function and secreted more insulin in response to high glucose stimuli (Supplementary Fig. 2D). Collectively, these data suggest that our hybrid implant neither interferes with glucose/insulin diffusion nor alters beta cell function entrapped inside them.

### 3.4 Viability and function of primary human islets within the hybrid implant

**Fig. 6:**
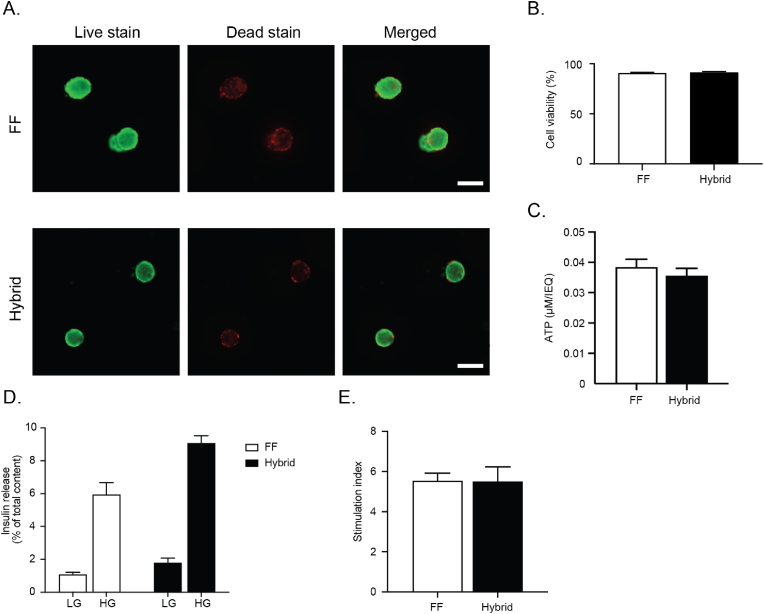
**Viability and function of human islets in the hybrid implant.** A) Representative live/dead staining images of microencapsulated human islets, free floating (FF) vs inside the hybrid implant (hybrid) at day 3 (scale bar = 200 μm). B) Percentage of cell viability from the live/dead staining images (mean ± SEM; n = 25 islets). C) The relative ATP amount in both conditions are depicted as per islet equivalent to compensate for variability in islet size indicative of the average number of metabolically active cells in both FF and hybrid at day 3 (mean ± SEM, n = 5, student-t-test ∗p < 0.05). D) Glucose stimulated insulin secretion (GSIS) of microencapsulated human islets, FF vs hybrid, at day 3. (LG = low glucose, 1.7; HG = high glucose, 16.7 mmol/l) E) Stimulation indices derived from the glucose stimulated insulin secretion test (mean ± SEM, n = 5, student-t-test ∗p < 0.05).

To demonstrate clinical translatability, human islets were encapsulated within alginate hydrogels and ∼50 microcapsules were seeded within the microwell array macrodevice. Microcapsules seeded within the macrodevice were observed to be evenly distributed with one microcapsule residing per microwell. Live/dead staining at day 3 of culture revealed that encapsulated human islets within the hybrid device maintained their viability (91.46 ± 1.11 %) similar to that of the free-floating controls (90.83 ± 1.00 %) (Fig. 6A and B). Similarly, no significant difference was found in the ATP levels between microencapsulated islets in the hybrid implant (0.036 ± 0.001 μM/IEQ) and free-floating controls (0.038 ± 0.00 μM/IEQ) (Fig. 6C). Next, encapsulated human islets were subjected to a GSIS assay to evaluate the functionality of the hybrid implant. Both hybrid implants and free-floating controls showed a characteristic low-high insulin release pattern in response to glucose stimuli, indicative of normal beta cell function (Fig. 6D). Microencapsulated human islets in the hybrid device exhibited a similar insulin secretory behavior in response to high glucose stimuli compared to free-floating controls with a stimulation index of 5.51 ± 0.72 and 5.54 ± 0.38 respectively (Fig. 6E). This data suggests again that the hybrid implant does not interfere with glucose/insulin diffusion and that the microcapsule entrapment within the implant did not affect beta cell function.

### 3.5 Prevascularization of the hybrid implant

**Fig. 7:**
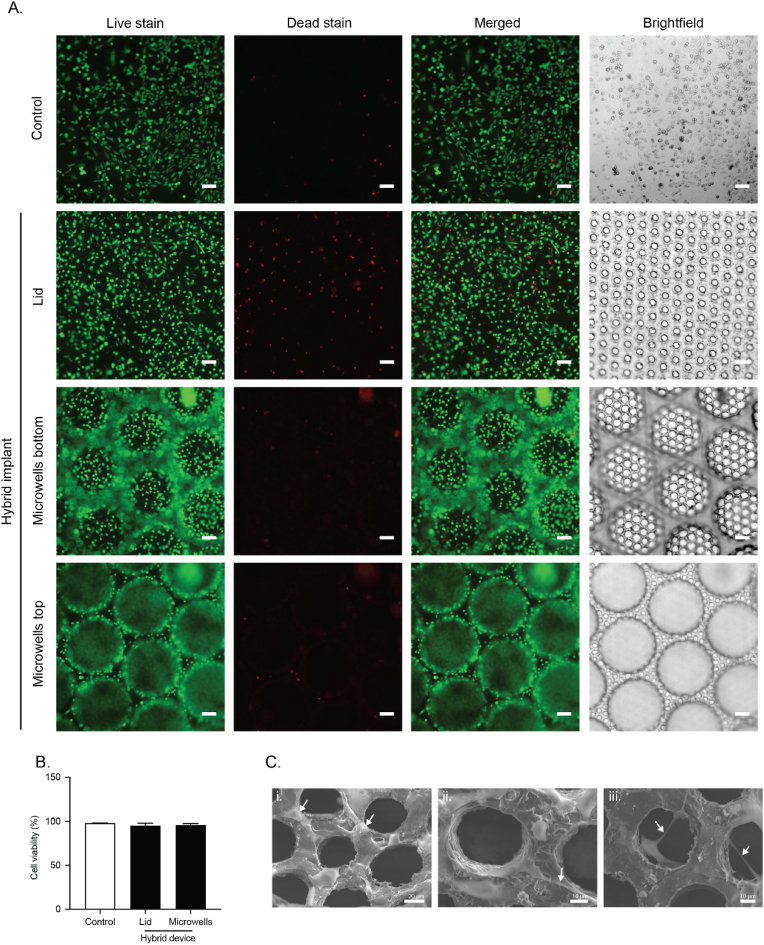
**Attachment and growth of HUVECs on the surface of the hybrid implant.** A) Representative live/dead staining images of HUVECs cultured on a gelatin-coated tissue culture plate (Control) or hybrid implant (microporous lid and bottom microwells PVDF film layers) for 14 days (scale bar = 100 μm). B) Percentage of cell viability from the live/dead staining images (mean ± SEM; n = 3 each 200–300 cells). C) Scanning electron micrographs of laser microdrilled PVDF microwell device surface seeded with HUVECs at 14 days of culture. White arrows indicate in (i) HUVECs spreading and growing over the PVDF film surface, in (ii) HUVECs sprouting pseudopodia-like structures and in (iii) HUVECs starting to bridge across the pores (scale bar = 10 μm).

To assess the prevascularization potential of the hybrid implant, HUVECs were cultured on the macrodevice films for 14 days and their attachment and viability were examined using scanning electron microscopy and live/dead staining, respectively. Live/dead staining results showed that HUVECs were able to adhere and proliferate on the surface of varied macrodevice components similar to the gelatin coated tissue culture plate controls (Fig. 7A). The viability of HUVECs adhered to macrodevice components (porous PVDF with (bottom) or without (lid) microwells) demonstrated a high percentage (≥95 %) similar to the gelatin-coated controls (Fig. 7B). Further, electron micrographs showed that several HUVECs had adhered to the surface of the macrodevice with cells spreading and growing through the pores and in some cases bridging some of the pores. Several instances of cell-to-cell contact and what looks like extended pseudopodia resembling primitive sprouting were observed (Fig. 7C). These results indicate that the open design and porous features of the macrodevice support the adherence and growth of HUVEC and thus the hybrid implant can potentially be prevascularized prior to implantation.

## 4 Discussion

In the present study, we report the design and fabrication of a hybrid implant using a biocompatible non-degradable polymer, PVDF. The hybrid implant features thin films of PVDF with unique microwell array structures supported with a PVDF support ring. We have demonstrated that these microwells were able to retain cell-alginate immunoprotective microcapsules separately providing optimal spacing in between and thus unhindered transport of nutrients and oxygen to the cells to maintain cell viability and function. Moreover, we have shown that endothelial cells were able to adhere and grow on the surface of the hybrid device films *in vitro*, providing the opportunity for prevascularizing the device prior to implantation thus increasing graft survival and function.

There are several challenges involved in the development of a combinatory hybrid implant for cell delivery applications. These include selecting an optimal biomaterial that prevents foreign body response, enables swift revascularization for cell nourishment, easy handling, and finally protects the encapsulated cells from host immune response. We report on a hybrid device using a macroporous PVDF-based envelope and ultrapure alginate microcapsules that combines favorable features of macro- and micro-encapsulation devices. The choice of PVDF was based on our recent biomaterial screening studies on beta cell survival and oxidative stress [28,29]. In those studies, multiple clinically used non-degradable biomaterials were tested on primary human islets and beta and alpha cell lines and showed that PVDF films induced relatively low levels of oxidative stress in the cells and had appropriate mechanical properties for the fabrication of macrodevices. An *in vivo* biocompatibility study by Klink et al. further showed that PVDF meshes induce low foreign body response, supporting their suitability for hernia repairs [30]. All these inherent properties of PVDF prompted us to produce the macroporous envelope (including microwells) of our hybrid implant from clinically graded PVDF. We used an automated film casting system to generate thin PVDF films (∼20 μm thick), followed by a femtosecond laser drilling technique to create a predefined pattern of pores (∼25 μm diameter) and finally, a microthermoforming technique to create a microwell array structure onto the porous films. The advantage of having thin and porous films is to support vascular ingrowth into the interior of the device to maximize mass transfer (of nutrients and oxygen) to the microencapsulated cells thereby promoting cell survival and function. The initially 25 μm diameter pores were stretched during microthermoforming of the PVDF films to reach a diameter ranging between 40 and 90 μm that is still suitable for vascular ingrowth [[31], [32], [33], [34]]. We have previously shown that a pore size of around 50 μm in a similar macroporous cell delivery construct allowed rapid revascularization of primary islets supporting mass transport of nutrients throughout the device [35]. Other parameters considered during the fabrication of the cell delivery device were its structural integrity and flexibility which are important for optimal surgical handling and tissue response. Previously, we found that macroporous devices made of thin films were difficult to handle and were prone to folding *in vivo* which could lead to suboptimal engraftment [24]. Therefore, we included an additional PVDF support ring during the assembly of the hybrid implant by ultrasonic welding around the rim to prevent folding. The support ring provides mechanical support for the microwells resulting in a slightly rigid device thereby enabling easy handling during loading of the microcapsules and cell culturing.

For the immunoprotective element of the hybrid implant, we used ultrapure high G (α-L-guluronic acid) alginate crosslinked with barium to form microcapsules using an air-driven droplet generator. Using barium ions instead of calcium or strontium yields microcapsules that are stronger and more stable with superior immunoprotective properties by being less permeable to molecules responsible for immune rejection such as immunoglobulin G [36]. The size of the microcapsule can play a role in its biocompatibility although there is no clear consensus regarding this. One study demonstrated that bigger alginate microcapsules (∼1.5 mm diameter) tend to induce less foreign body response compared to smaller-sized counterparts [37], while we and others showed similar biocompatibility with smaller (∼400–700 μm) microcapsules [[38], [39], [40], [41], [42]]. For our hybrid device, we generated microcapsules of ∼500 μm diameter to increase the cell loading capacity while maintaining the macrodevice size small. Maximizing the cell loading capacity can result in too densely packed microcapsules whereby the diffusion of oxygen and nutrients might decrease moving towards the core region of the macrodevice resulting in a loss of cell function over time [43]. The dense packing of microcapsules is resolved by a unique feature included in the hybrid implant, namely the microwell array structure, where each microwell (average diameter of 818.2 ± 21 μm and a depth of 747.8 ± 16.7 μm) is large enough to capture a single microcapsule of ∼500 μm. These microwell structures physically separate and homogenously distribute the microcapsules throughout the device thereby potentially maximizing mass transport and allowing for revascularization around the microcapsules. The confinement of microcapsules into separate porous microwells will potentially enhance the nourishment of cells thereby promoting cell survival and function. Another challenge with microcapsules is the ability to easily retrieve them. Incomplete retrieval can cause serious safety concerns especially when stem cell-derived cells are used due to possible teratoma formation [44,45]. Common retrieval techniques such as aspiration and lavage are time-consuming and do not necessarily ensure complete retrieval of all microcapsules given their large number and small size enabling them to spread over organ cavities at the site of transplantation, such as the peritoneal space [46,47]. With our hybrid implant, complete retrieval of microcapsules is feasible as they remain confined in the microwells in between two macroporous layers thereby mitigating any safety concerns.

To evaluate the capability of the hybrid implant to sustain cell viability and function, as a proof-of-concept, rodent pseudoislets or primary human islets were encapsulated in alginate microcapsules and subsequently seeded into the macrodevice. Beta cells within islets display a high metabolic activity and therefore require sufficient access to oxygen and nutrients to survive and function [48,49]. Microencapsulated islets entrapped in the hybrid implant showed no significant difference in ATP levels and viability (live/dead) compared to free-floating encapsulated islets. Functionally, islets in the hybrid implant showed a comparable glucose-stimulated insulin secretion to that of the free-floating counterpart. These data suggest that the hybrid implant with its thin and porous films allows for unhindered mass transport of relevant compounds (oxygen, nutrients, glucose, and endocrine hormones) to and from the microencapsulated islets without causing hypoxia. Furthermore, the barium alginate microcapsules of the hybrid implant can provide immunoprotection to the islets as demonstrated in several studies [[50], [51], [52], [53]].

Device prevascularization is an additional strategy that can be employed to boost oxygen and nutrient supply to encapsulated cells after implantation thereby promoting cell survival and function. Studies have shown that devices preloaded with mesenchymal stem cells, platelet-rich-plasma or HUVEC resulted in a dense vascular network by promoting neoangiogenesis and anastomoses and improved graft survival when transplanted in the subcutaneous space [[54], [55], [56]]. In this study, we tested if the hybrid implant supports the growth of HUVEC on its surface. The cells have spread and grown through the pores of the macrodevice (40–90 μm in diameter) which could allow for vascular ingrowth and rapid revascularization [[31], [32], [33]]. This result indicates the prevascularization potential of the hybrid implant using endothelial cells prior to transplantation to enhance engraftment and function, especially in poorly vascularized sites such as the subcutaneous space. Future studies will explore the performance of encapsulated islet-loaded prevascularized microwell hybrid implants in diabetic animal models.

## 5 Conclusion

In summary, the macrodevice with its patterned microwell array structures made from thin and porous clinical grade PVDF combined with microencapsulation can support the survival and function of encapsulated cells (both allogenic or xenogenic) by providing nutrients, oxygen as well as a safe immune privileged space. Furthermore, the device design is scalable as we have built an in-house software application that allows for extrapolation of the design to human-sized devices with realistic clinically relevant dimensions. The unique architecture of our hybrid implant combined with its easy handling, scalability, retrievability, immunoprotectivity, and prevascularization potential makes it an attractive choice as a potential cell delivery device for a wide range of cell therapy applications including stem cells.

## CRediT authorship contribution statement

**Adam Stell:** Writing – review & editing, Writing – original draft, Visualization, Methodology, Investigation, Formal analysis, Data curation, Conceptualization. **Vijayaganapathy Vaithilingam:** Writing – review & editing, Writing – original draft, Visualization, Methodology, Investigation, Formal analysis, Data curation, Conceptualization. **Sami G. Mohammed:** Writing – review & editing, Writing – original draft, Visualization, Methodology, Investigation, Formal analysis, Data curation, Conceptualization. **Rick H.W. de Vries:** Methodology, Investigation, Formal analysis. **Denise F.A. de Bont:** Methodology, Investigation, Formal analysis. **Eelco J.P. de Koning:** Resources. **Aart A. van Apeldoorn:** Writing – review & editing, Visualization, Supervision, Project administration, Funding acquisition, Conceptualization.

## Declaration of competing interest

The authors declare the following financial interests/personal relationships which may be considered as potential competing interests:Aart van Apeldoorn is the founder and shareholder of Lighthouse Biomedical B.V. a start-up company aiming to commercialize beta cell delivery devices in the future. If there are other authors, they declare that they have no known competing financial interests or personal relationships that could have appeared to influence the work reported in this paper.

## Data Availability

Data will be made available on request.
