# Supplementary material for: A hybrid implant combining a macroporous device with immunoprotective microcapsules for cell therapy applications: A conceptual in vitro study
Source: Mater Today Bio. 2025 Feb 27;31:101574. doi: 10.1016/j.mtbio.2025.101574 (PMC11931250; doi:10.1016/j.mtbio.2025.101574)
Supplement: Multimedia component 1 [file mmc2.docx]

**Supplementary Material**

**A Hybrid implant combining a macroporous device with immunoprotective microcapsules for cell therapy applications: A conceptual in vitro study**

Adam Stell^a^**^*^**, Vijayaganapathy Vaithilingam^a^**^*^**, Sami G. Mohammed^a*^, Rick H.W. de Vries^a^, Denise F.A. de Bont^a^, Eelco J.P. de Koning^b, c^, Aart A. van Apeldoorn^a, d^

^a^ Department of Cell Biology–Inspired Tissue Engineering (cBITE), MERLN Institute for Technology Inspired Regenerative Medicine, Maastricht, The Netherlands

^b^ Department of Internal Medicine, Leiden University Medical Center (LUMC), P.O. Box 9600, 2300 RC, Leiden, the Netherlands

^c^ LUMC Transplantation Center, Leiden University Medical Center, Leiden, the Netherlands.

^d^ Lighthouse Biomedical B.V., Maastricht, Netherlands.

***** Authors contributed equally to this work**

**Correspondence to**

**Aart A. van Apeldoorn**

Department of Cell Biology–Inspired Tissue Engineering,

MERLN Institute, Maastricht University,

PO Box 616, 6200 MD Maastricht, The Netherlands.

e-mail: [a.vanapeldoorn@maastrichtuniversity.nl](mailto:a.vanapeldoorn@maastrichtuniversity.nl)

Telephone: 0031 43 388 2222


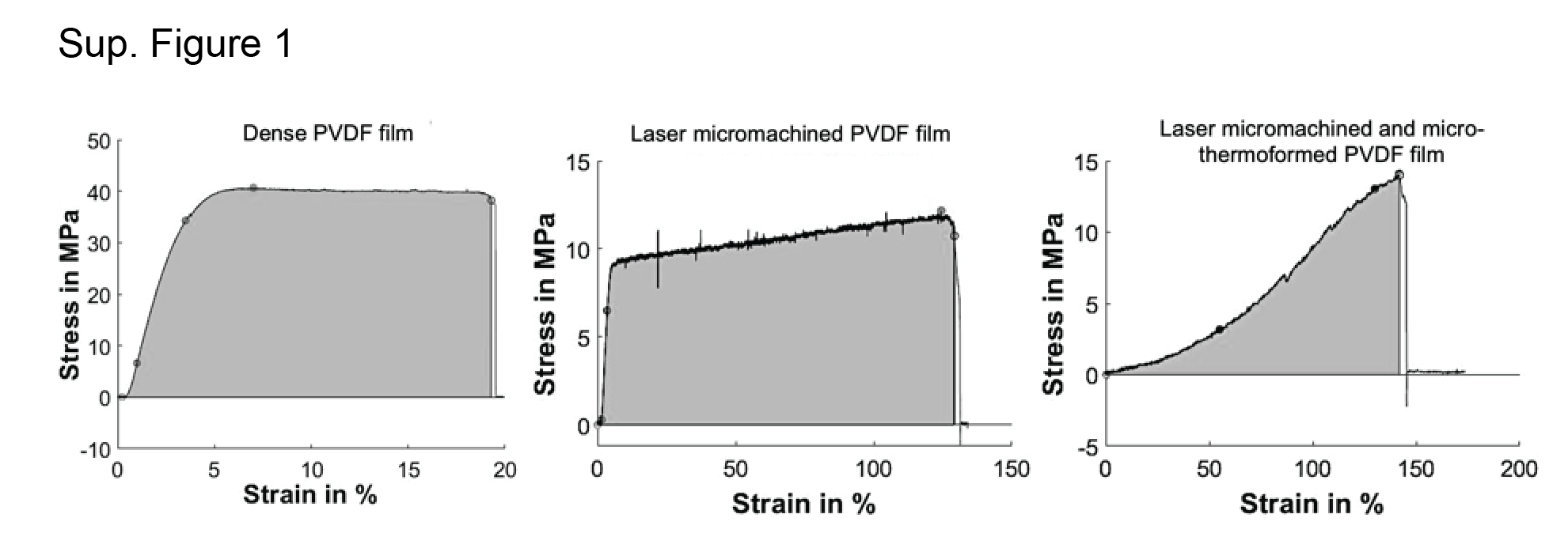


**Supplementary figure 1: Stress-strain curves of PVDF films after different processing steps.** The slope of stress-strain curves shows clear changes after each step. The slope of the curve in thermoformed films is less steep compared to the other films (dense PVDF films and laser micromachined PVDF films) indicative of a considerable decrease of the Young’s modulus.

**Supplementary movie 1. Handling and testing integrity of the assembled device.** The assembled device was held easily with forceps. Furthermore, it remained intact and retained its structure after being folded and released.

**Supplementary movie 2. Method of loading the device with microencapsulated cells.** Microcapsules are resuspended in 200 µl culture medium and loaded into the device via the inlet using a wide-pore pipette tip. While seeding, the pipette tip is slowly moved from side to side to distribute the microcapsules evenly throughout the device.


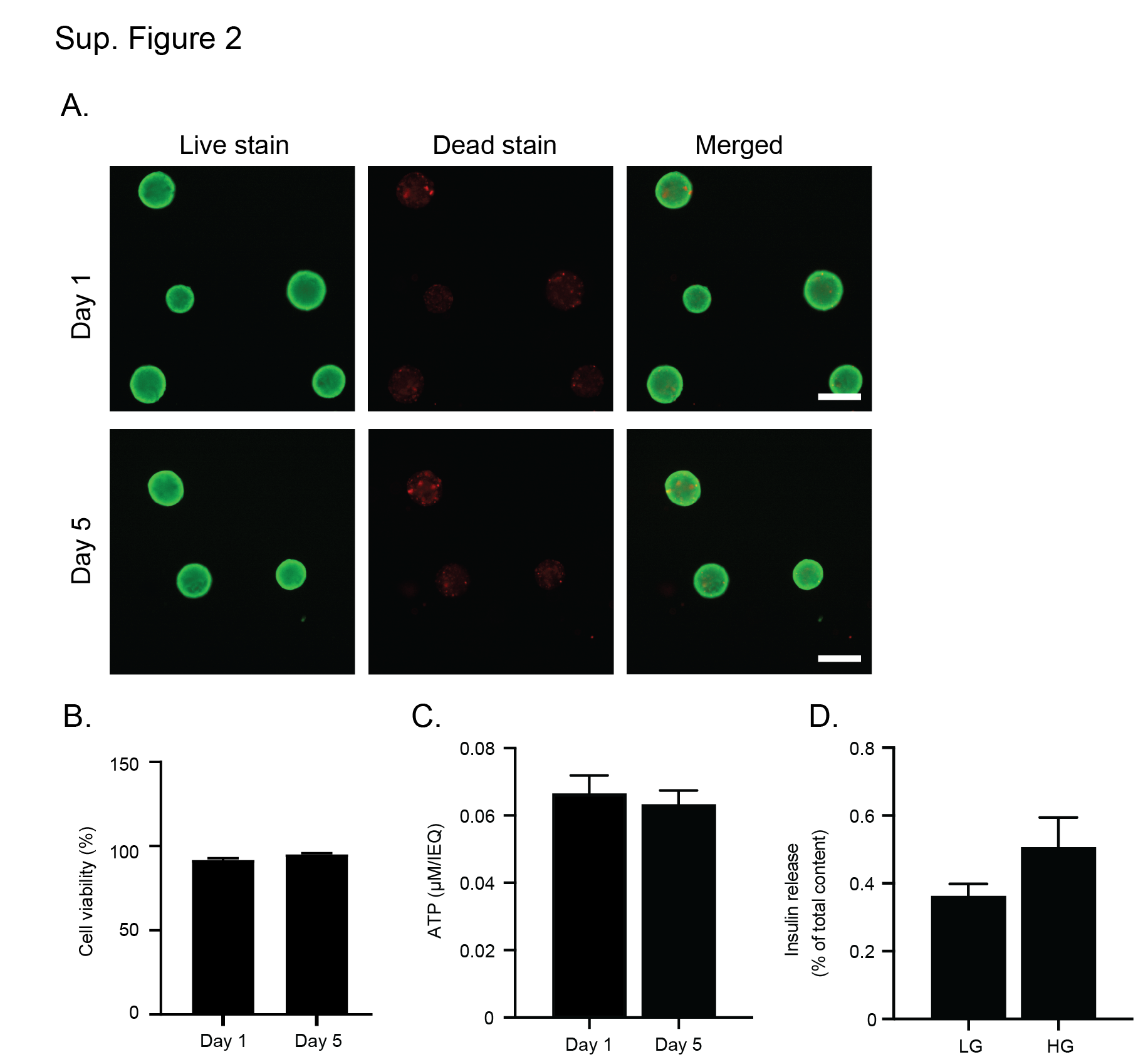


**Supplementary figure 2. Viability and function of MIN6 pseudoislets in the hybrid implant.** A) Live/dead staining of MIN6 pseudoislets cultured in the hybrid implant at day 1 and day 5 after seeding (scale bars = 200 μm). B) Percentage of cell viability from the live/dead staining images (mean ± SEM; n = 25 islets). C) The relative ATP amount in both conditions are depicted as per islet equivalent to compensate for variability in islet size indicative of the average number of metabolically active cells within the hybrid implant on both days 1 and 5 (mean ± SEM, n=6, student-t-test * p<0.05) respectively. D) Glucose stimulated insulin secretion (GSIS) of microencapsulated MIN6 pseudoislets in the hybrid implant at day 5 post-seeding. (LG= low glucose, 1.7; HG= high glucose, 16.7 mmol/l) (mean ± SEM, n=3).
